# Blood Transfusions in Total Hip and Knee Arthroplasty: An Analysis of Outcomes

**DOI:** 10.1155/2014/623460

**Published:** 2014-01-21

**Authors:** Thomas Danninger, Rehana Rasul, Jashvant Poeran, Ottokar Stundner, Madhu Mazumdar, Peter M. Fleischut, Lazaros Poultsides, Stavros G. Memtsoudis

**Affiliations:** ^1^Department of Anesthesiology, Hospital for Special Surgery, Weill Medical College of Cornell University, 535 East 70th Street, New York, NY 10021, USA; ^2^Department of Public Health, Division of Biostatistics and Epidemiology, Weill Medical College of Cornell University, 402 East 67th Street, New York, NY 10065, USA; ^3^Department of Anesthesiology, Perioperative Medicine and Critical Care Medicine, Paracelsus Medical University, Muellner Hauptstraße 48, 5020 Salzburg, Austria; ^4^Department of Anesthesiology, Weill Medical College of Cornell University, 402 East 67th Street, New York, NY 10065, USA; ^5^Department of Orthopedic Surgery, Hospital for Special Surgery, Weill Medical College of Cornell University, 535 East 70th Street, New York, NY 10021, USA

## Abstract

*Background.* Various studies have raised concern of worse outcomes in patients receiving blood transfusions perioperatively compared to those who do not. In this study we attempted to determine the proportion of perioperative complications in the orthopedic population attributable to the use of a blood transfusion. *Methods.* Data from 400 hospitals in the United States were used to identify patients undergoing total hip or knee arthroplasty (THA and TKA) from 2006 to 2010. Patient and health care demographics, as well as comorbidities and perioperative outcomes were compared. Multivariable logistic regression models were fitted to determine associations between transfusion, age, and comorbidities and various perioperative outcomes. Population attributable fraction (PAF) was determined to measure the proportion of outcome attributable to transfusion and other risk factors. *Results.* Of 530,089 patients, 18.93% received a blood transfusion during their hospitalization. Patients requiring blood transfusion were significantly older and showed a higher comorbidity burden. In addition, these patients had significantly higher rates of major complications and a longer length of hospitalization. The logistic regression models showed that transfused patients were more likely to have adverse health outcomes than nontransfused patients. However, patients who were older or had preexisting diseases carried a higher risk than use of a transfusion for these outcomes. The need for a blood transfusion explained 9.51% (95% CI 9.12–9.90) of all major complications. *Conclusions.* Advanced age and high comorbidity may be responsible for a higher proportion of adverse outcomes in THA and TKA patients than blood transfusions.

## 1. Introduction

Over the last decades, a growing body of literature has been published in which authors report worse outcomes in patients receiving blood transfusions compared to those that do not in various medical settings [1–8]. However, not all reports have come to the same conclusion [9] and considerable controversy persists regarding cause and effect. In this context, relationships between the need for blood transfusions and other confounders that may contribute to increased risk of adverse outcomes may exist [9].

While most data available stem from institutional studies performed in a controlled setting in academic centers, there is a lack of population based data to elucidate the issue of blood management in patients undergoing either THA or TKA. Therefore, we used a large nationwide database to (1) analyze characteristics of patients either receiving blood transfusion or not after THA or TKA, (2) compare the risk of using a transfusion to other risks of perioperative outcomes with and without adjustments, and (3) determine the impact of blood transfusions on the population level with respect to complication rates using population attributable fraction (PAF). PAFs provide additional information beyond measures on the strength of association: a risk factor may have a high odds ratio for perioperative complications; however, on the population level its attributable risk is limited if the risk factor is very rare; PAFs account for this [10].

We hypothesized that patients receiving blood transfusions in the perioperative phase were older and had higher comorbidity burden compared to those that did not and that the risk of having a transfusion is reduced when adjusting for advanced age and comorbidities. We further hypothesized the PAF for adverse events associated with blood transfusions to be substantial, but smaller than the PAF associated with increased comorbidity burden or advanced age.

## 2. Material and Methods

### 2.1. Study Design and Data Source

We conducted a retrospective cohort study using administrative data from the Premier Perspective database (Premier Inc., Charlotte, NC) collected from January 2006 to September 2010. (http://www.premierinc.com/). This database features information from approximately 400 acute care hospitals located throughout the United States. Data include specific information on present diagnoses or procedures as well as complete billing and coding details. During the data collection and distribution process, rigorous and standardized validation screening is carried out to ensure data quality [11]. Due to the fact that data is deidentified and meets the criteria of the Health Insurance Portability and Accountability Act to protect personal information, this study was exempt from the consent requirements by our Institutional Review Board.

### 2.2. Study Sample

The study cohort consisted of all cases in the premier system indicating that a patient received either THA or TKA using the International Classification of Diseases-9th revision-Clinical Modification (ICD-9-CM), procedure codes 81.51 and 81.54, respectively.

### 2.3. Study Variables

Patient related demographics analyzed included age, gender, race (White, Black, Hispanic, and other), type of insurance (commercial, Medicaid, Medicare, uninsured, and other), and type of admission (emergent, urgent, elective, and other). Healthcare related variables included hospital location (urban, rural), hospital size (≤299, 300– 499, ≥500 beds) and teaching status. Procedure related variables were type of anesthesia (general, neuraxial, neuraxial and general, and other), peripheral nerve block use, type of surgery (THA or TKA), year of procedure, cost of hospitalization, and length of hospitalization. Type of anesthesia and use of a peripheral nerve block were identified using billing records. Use of a blood transfusion was identified by the ICD-9-CM codes 99.00–99.09.

We also included individual comorbidities from the Deyo-Charlson and Elixhauser groups [12, 13]. The groups were originally used to predict 1-year mortality (Charlson) or length of stay, hospital charges, and in-hospital death (Elixhauser). They were identified using ICD-9 codes as previously reported. (http://www.hcup-us.ahrq.gov/toolssoftware/comorbidity/comorbidity.jsp). A measure of overall comorbidity burden was determined using the method by Deyo et al. [12]. In addition, sleep apnea and pulmonary hypertension, defined using ICD-9 codes (Table 6) were considered because these comorbidities were deemed important although not included in either index [14].

### 2.4. Outcome Measures

Complication variables included: major cardiac complications (acute myocardial infarction and other cardiac related complications), major pulmonary complications (pulmonary embolism, pneumonia, and other pulmonary complications), deep venous thrombosis, cerebrovascular events, infections, acute renal failure, gastrointestinal complications, and 30-day mortality. A combined major complications variable, which includes having any of the previous complications listed, was also created. Complication variables were defined using ICD-9 and CPT codes (Table 7). 30-day mortality includes all cases where a patient died in current or subsequent admissions within 30 days. Additionally, critical care service utilization (CCS), defined using billing records, was also measured.

### 2.5. Statistics

Patients were characterized by receipt of a blood transfusion. Significance was assessed using Chi-square tests and *t*-tests of means for categorical and continuous variables, respectively. Median and interquartile ranges (IQR) were reported for length and cost of hospitalization because of their skewed distribution. Significance for these variables was measured using the Mann-Whitney rank sum test.

Univariable and multivariable logistic regression models were fitted to determine the association between the use of a transfusion and the following outcomes: major combined complications, cardiac complications, pulmonary complications, renal failure, 30-day mortality, and CCS utilization. Adjustments of all demographic, healthcare related, and procedure related variables were included in models to account for patient and practice patterns. Comorbidity variables (diabetes, renal disease, cerebrovascular disease, peripheral vascular disease, cancer, obesity, pulmonary hypertension, and sleep apnea) were included in the multivariable models after univariable Chi-square tests showed significance (*P* < 0.05). Although obesity was not significantly associated with 30-day mortality, it was deemed clinically relevant and included. Model discrimination, indicating how well the model differentiates observed data at different levels of the outcome, was measured using the area under the receiving operator characteristic curve (*c*-statistic) [15]. A model with a *c*-statistic > 0.7 indicates acceptable discrimination.

PAF is the proportion of the outcome attributable to individuals living with risk compared to those without [10]. The standard PAF calculation is as follows:

(1) $$\begin{array}{c} \text{PAF}\%=\left[\frac{\left(P*\left(RR-1\right)\right)}{\left(1+\left(P*\left(RR-1\right)\right)\right)}\right]*100.\; \end{array}$$

From this formula PAF estimations are expressed as “the percentage or proportion of outcome accounted for” by the risk factor involved. RR stands for relative risk and *P* for the prevalence of the risk factor in the studied population.

PAF estimates from the above formula are subject to limitations, in particular in case of confounding [16]. We therefore computed covariate-adjusted PAF using multivariable logistic models according to the method by Greenland and Drescher [15] for cohort and cross-sectional studies. PAF and 95% confidence intervals (CI) were measured for transfusion. PAFs for nonmodifiable risks were also included to understand their contribution to the proportion of adverse health events in this population.

Analyses were performed in SAS version 9.3 statistical software (SAS Institute, Cary, NC, USA). Population attributable fraction was calculated using the *punaf *package in the STATA version 12.1 statistical package (StataCorp, College Station, TX, USA) [17].

## 3. Results

We identified 530,089 entries for THA or TKA. Of those, 100,352 (18.9%) received a blood transfusion.  Table 1 shows patient and health care system related information by transfusion status. Patients with the need for a transfusion were on average older (mean age 68.9 (SD = 11.4) versus 65.2 (SD = 11.1) years, *P* < 0.001) and showed a higher comorbidity burden (mean Deyo Index 0.71 (SD = 1.0) versus 0.60 (SD = 0.9), *P* < 0.001). Furthermore, rates of patients receiving a blood transfusion were higher among females and minorities (Table 1). Patients receiving blood transfusions showed significantly higher rates of individual comorbidities (Table 2).

In addition, patients receiving blood transfusions showed significantly higher rates for combined major complications (19.1% versus 11.2%, *P* < 0.0001) as well as higher rates of 30-day mortality, use of mechanical ventilation, and critical care services (Table 3). Moreover, transfused patients had a significantly increased median length of hospital stay (3 [IQR: 3-4] days versus 4 [IQR: 3–5] days, *P* < 0.0001); further, median cost of care was higher in the group of patients requiring a transfusion (USD 16,998 [IQR: USD 13,712-USD 21,797] versus USD 14,678 [IQR: USD 12,109-USD 18,093], *P* < 0.0001).

The unadjusted (univariate) regression models showed that transfused patients were more likely to have adverse health outcomes than the patients who were not transfused. However, after adjustment, we found lower odds ratios (OR), which indicates presence of confounding due to demographics and comorbidities. For almost all outcomes, higher odds ratios were found when patients were older or had a history of renal disease, cerebrovascular disease, or pulmonary hypertension. Odds ratios, 95% confidence intervals (CI) for the unadjusted and adjusted models are shown in Table 4. Population attributable fractions for several risk factors and outcomes are shown in Table 5. The proportion of combined complications potentially attributable to blood transfusion in this patient population was 9.51% (95% CI: 9.12%–9.90%). The PAF for advanced age and comorbidity burden varied from 11.37% to 58.02%, and 12.42% to 32.77%, respectively, with the PAF for age being higher than the PAF for comorbidity burden in combined major complications, major cardiac complications, and 30-day mortality.

## 4. Discussion

Our analysis of more than half a million patients between 2006 and 2010 showed that approximately 19% (*n* = 100, 352) of all patients undergoing THA and TKA required a perioperative blood transfusion.

The group of patients receiving blood after THA or TKA was on average older and had a higher comorbidity burden as well as significantly worse outcomes compared to the group that was not transfused. In particular, this related to a higher incidence of major cardiac and pulmonary complications, more frequent use of mechanical ventilation, and a higher rate of critical care service utilization. Therefore, our findings based on the analysis of data from over 400 US hospitals are consistent with studies that have previously been published [1–8]. However, we were able to determine that the risk of blood transfusions was lower than the risk of advanced age and comorbidity burden. Further, the proportion of complications attributable to blood transfusions was lower than that attributable to other factors such as comorbidity burden and advanced age in the context of combined major complications, major cardiac complications, and 30-day mortality.

We identified a number of differences in the characteristics of patients receiving blood transfusions versus those that did not. Patients receiving a transfusion were older, more likely female, and had a higher comorbidity burden. Minorities had higher rates of needing a transfusion also. Advanced age and higher comorbidity burden have been associated with a decrease in end organ reserve [18] and may thus explain the decision of physicians to transfuse patients more readily in an attempt to maintain oxygen delivery in a presumably more vulnerable population.

The need for higher transfusion rates among females may have its cause in the generally lower circulating blood volume and baseline hematocrit compared to their male counterparts [19].

The finding that blood transfusions among racial minorities were used more frequently was surprising and needs further investigation. Although disparities in health care have been described in the past, they are usually associated with an underutilization of resources [20, 21].

We found that blood transfusions were associated with increased incidence and risk for complications and increased resource utilization. This was true for cardiac and pulmonary complications, acute renal failure, and 30-day mortality as well as for the utilization of mechanical ventilation and critical care services. However, the occurrence of major complications or the extended use of resources cannot be causally linked to a single intervention, specifically blood transfusion, during the perioperative course of a surgical procedure. Instead, it may remain more important to take other various factors, like age and/or preexisting comorbidity burden, into account.

We found that the use of a blood transfusion could explain approximately 10% of combined adverse events. In contrast, the PAF of presence of comorbidities was higher (13.4%) as was the PAF of advanced age (34.7%) Although a significant proportion of complications may be related to transfusions, either the reasons for or the consequences thereof, the contribution of other variables to these outcomes should be considered as well. This analysis is especially useful as it may represent an attempt to statistically account for the fact that sicker and older patients may be at increased risk for requiring blood transfusions per se. Thus, our results may be used to more differentially address the issue frequently raised that blood transfusions may just be a surrogate marker for variables indicating increased morbidity.

A number of limitations of our study have to be addressed. Many are related to the analysis of secondary data from large administrative databases. One such limitation affects the inability to identify causal relationships. Further, important information surrounding the blood transfusions themselves is not available, including the amount of blood loss, the number of units administered, and information regarding the hematocrit value that preceded the decision as well as the clinical appearance of the patient. Secondly, only events that occur within the index hospitalization can be investigated; data on postdischarge events (except 30-day mortality) are not available.

Nevertheless, databases, like the one used, provide access to a large number of patients from a wide range of clinical practice settings and therefore represent a rare opportunity to investigate topics in the context of real world practice. Despite the rigorous controls before entering data into the database, it must also be mentioned that there is a possibility of coding errors when using ICD-9 codes. However, it is likely to be evenly distributed across the whole dataset and resulting bias may therefore be of reduced relevance.

In conclusion, in this study of population based data examining patients undergoing THA or TKA, we found that patients receiving blood transfusions were older and had a higher comorbidity burden. Further, higher rates and independent risk for adverse outcomes and increased resource utilization was found in this group. Although approximately 9.5% of all complications could be attributed to blood transfusion related factors, comorbidity burden, and advanced age were able to explain a higher proportion of adverse events. Therefore, other patient variables should be taken into account more critically when interpreting risk of adverse outcomes in patients receiving blood transfusions.

## Figures and Tables

**Table 1:** Patient-, healthcare-, and procedure-related characteristics are listed in this table, characterized by transfusion.

Characteristic	Category	Transfusion	No transfusion	*P* value
		*N* (%)	*N* (%)	
Study population		100,352 (18.93)	429,737 (81.07)	

Patient related				
Age category	<45	2,324 (2.32)	13,616 (3.17)	<0.001
	45–54	8,938 (8.91)	58,684 (13.66)	
	55–64	21,830 (21.75)	126,386 (29.41)	
	65–74	31,836 (31.72)	136,396 (31.74)	
	75–99	35,424 (35.3)	94,655 (22.03)	
Mean age (SD)		68.87 (SD = 11.4)	65.24 (SD = 11.1)	
Gender	Female	71,804 (71.55)	253,074 (58.89)	<0.001
	Male	28,548 (28.45)	176,663 (41.11)	
Race	White	69,573 (69.33)	325,498 (75.74)	<0.001
	Black	8,196 (8.17)	28,001 (6.52)	
	Hispanic	3,093 (3.08)	8,839 (2.06)	
	Other	19,490 (19.42)	67,399 (15.68)	
Deyo index category	0	59,145 (58.94)	276,833 (64.42)	<0.001
	1	19,116 (19.05)	70,197 (16.34)	
	2	15,873 (15.82)	61,933 (14.41)	
	≥3	6,218 (6.20)	20,774 (4.83)	
Mean deyo index (SD)		0.71 (SD = 1.0)	0.60 (SD = 0.9)	

Healthcare related				
Hospital location	Rural	11,040 (11)	42,403 (9.87)	<0.001
	Urban	89,312 (89)	387,334 (90.13)	
Hospital bed size	≤299	31,548 (31.44)	140,010 (32.58)	<0.001
	300–499	34,932 (34.81)	173,293 (40.33)	
	≥500	33,872 (33.75)	116,434 (27.09)	
Hospital teaching status	No	61,861 (61.64)	249,435 (58.04)	<0.001
	Yes	38,491 (38.36)	180,302 (41.96)	

Procedure related				
Year of procedure	2006	19,097 (19.03)	78,641 (18.3)	<0.001
	2007	20,705 (20.63)	84,549 (19.67)	
	2008	22,348 (22.27)	89,248 (20.77)	
	2009	22,703 (22.62)	100,829 (23.46)	
	2010	15,499 (15.44)	76,470 (17.79)	
Type of anaesthesia	Neuraxial	6,644 (6.62)	33,390 (7.77)	<0.001
	General	54,868 (54.68)	239,425 (55.71)	
	Neuraxial/general	8,635 (8.6)	40,786 (9.49)	
	Other	30,205 (30.1)	116,136 (27.02)	
Peripheral nerve block	Yes	93,587 (93.26)	394,244 (91.74)	<0.001
	No	6,765 (6.74)	35,493 (8.26)	
Type of procedure	THA	40,532 (40.39)	132,418 (30.81)	<0.001
	TKA	59,820 (59.61)	297,319 (69.19)	

CI: confidence interval and SD: standard deviation.

**Table 2:** The prevalence of selected comorbidities is listed in this table characterized by transfusion.

Comorbidity	Transfusion	No transfusion	*P* value
	*N* (%)	*N* (%)	
Myocardial infarction	4,267 (4.25)	15,235 (3.55)	<0.001
Peripheral vascular disease	2,446 (2.44)	6,798 (1.58)	<0.001
Cerebrovascular disease	392 (0.39)	842 (0.2)	<0.001
Dementia	176 (0.18)	326 (0.08)	<0.001
COPD	15,420 (15.37)	60,447 (14.07)	<0.001
Rheumatic disease	5,312 (5.29)	15,411 (3.59)	<0.001
Mild liver disease	479 (0.48)	858 (0.20)	<0.001
Severe liver disease	135 (0.13)	127 (0.03)	<0.001
Diabetes	18,734 (18.67)	73,690 (17.15)	<0.001
Complicated diabetes	1,561 (1.56)	4,157 (0.97)	<0.001
Renal disease	92 (0.09)	174 (0.04)	<0.001
Cancer	2,636 (2.63)	6,702 (1.56)	<0.001
Hypertension	61,924 (61.71)	262,215 (61.02)	<0.001
Complicated hypertension	6,753 (6.73)	13,587 (3.16)	<0.001
Pulmonary hypertension	1,104 (1.10)	2,137 (0.50)	<0.001
Deficiency anemia	35,186 (35.06)	81,132 (18.88)	<0.001
Pulmonary circulation disorder	3,067 (3.06)	7,103 (1.65)	<0.001
Fluid and electrolyte disorders	20,553 (20.48)	48,526 (11.29)	<0.001
Psychoses	3,130 (3.12)	9,583 (2.23)	<0.001
Sleep apnea	6,394 (6.37)	37,852 (8.81)	<0.001
Obesity	15,545 (15.49)	78,807 (18.34)	<0.001

**Table 3:** The incidence of complications, mortality, and resource utilization, characterized by transfusion usage.

Complication	Transfusion	No transfusion	*P* value
	*N* (%)	*N* (%)	
Combined major complications	19,127 (19.06)	48,214 (11.22)	<0.001
Major cardiac complications	8,721 (8.69)	25,785 (6)	<0.001
Acute myocardial infarction	719 (0.72)	675 (0.16)	<0.001
Cardiac (Non-MI)	8,441 (8.41)	25,516 (5.94)	<0.001
Major pulmonary complications	3,274 (3.26)	5,803 (1.35)	<0.001
Pulmonary embolism	733 (0.73)	1,442 (0.34)	<0.001
Pulmonary complications	1,368 (1.36)	2,143 (0.5)	<0.001
Pneumonia	1,706 (1.7)	2,872 (0.67)	<0.001
Deep venous thrombosis	958 (0.95)	1,973 (0.46)	<0.001
Cerebrovascular event	222 (0.22)	384 (0.09)	<0.001
All infections	7,063 (7.04)	14,574 (3.39)	<0.001
Acute renal failure	3,488 (3.48)	4,498 (1.05)	<0.001
Gastrointestinal complication	1,168 (1.16)	2,910 (0.68)	<0.001
Mortality (30 day)	288 (0.29)	513 (0.12)	<0.001
Mechanical ventilation	1,213 (1.21)	2,592 (0.6)	<0.001
Critical care services admission	4,975 (4.96)	12,385 (2.88)	<0.001

MI: myocardial infarction.

**Table 4:** This table shows the unadjusted and adjusted logistic regression analyses measuring the association of use of transfusion, age, and presence of comorbidities with various outcomes.

Effect	Outcome					
	Combined complications	Cardiac complications	Pulmonary complications	Renal failure	ICU utilization	30-Day mortality
	Odds ratio (95% CI)	Odds ratio (95% CI)	Odds ratio (95% CI)	Odds ratio (95% CI)	Odds ratio (95% CI)	Odds ratio (95% CI)
Transfusion (unadjusted)	1.86 (1.83–1.90)*	1.49 (1.45–1.53)*	2.46 (2.36–2.57)*	3.40 (3.26–3.56)*	1.76 (1.70–1.82)*	2.41 (2.08–2.78)*
Transfusion	1.65 (1.62–1.69)*	1.28 (1.25–1.32)*	2.29 (2.19–2.40)*	3.22 (3.08–3.38)*	1.78 (1.72–1.84)*	1.74 (1.50–2.03)*
Age group (ref: ≤45 years)						
45–54	1.23 (1.14–1.33)*	1.68 (1.42–1.99)*	1.21 (1.00–1.45)**	1.37 (1.10–1.72)**	1.08 (0.97–1.21)	1.82 (0.76–4.33)
55–64	1.78 (1.65–1.92)*	3.29 (2.80–3.87)*	1.50 (1.26–1.78)*	2.04 (1.65–2.52)*	1.11 (1.00–1.24)***	2.42 (1.05–5.56)***
65–74	2.54 (2.35–2.74)*	6.62 (5.62–7.79)*	1.54 (1.29–1.84)*	2.16 (1.74–2.68)*	1.13 (1.01–1.26)**	3.07 (1.33–7.12)**
75–99	4.35 (4.03–4.69)*	13.85 (11.76–16.31)*	2.16 (1.80–2.59)*	3.30 (2.66–4.11)*	1.64 (1.47–1.83)*	7.86 (3.41–18.14)*
Diabetes	1.22 (1.20–1.25)*	1.15 (1.15–1.18)*	1.11 (1.06–1.17)*	1.64 (1.56–1.72)*	1.14 (1.09–1.18)*	1.28 (1.09–1.52)**
Renal disease	2.61 (1.98–3.44)*	1.88 (1.27–2.77)*	3.36 (2.06–5.47)*	3.35 (2.08–5.40)*	3.09 (2.06–4.64)*	2.98 (0.73–12.21)
Cerebrovascular disease	2.09 (1.84–2.37)*	2.14 (1.3–2.50)*	2.25 (1.75–2.89)*	1.61 (1.21–2.15)*	1.98 (1.61–2.43)*	2.71 (1.53–4.79)*
Peripherovascular disease	1.63 (1.55–1.71)*	1.67 (1.58–1.78)*	1.48 (1.32–1.66)*	1.68 (1.51–1.88)*	1.73 (1.59–1.88)*	1.75 (1.31–2.35)*
Cancer	1.37 (1.30–1.45)*	1.17 (1.09–1.26)*	1.71 (1.53–1.92)*	1.48 (1.31–167)*	1.77 (1.62–1.93)*	2.03 (1.51–2.73)*
Obesity	1.36 (1.33–1.39)*	1.22 (1.18–1.26)*	1.49 (1.41–1.57)*	2.19 (2.08–2.31)*	1.48 (1.42–1.53)*	1.44 (1.18–1.75)*
Pulmonary hypertension	4.12 (3.83–4.43)*	4.88 (4.51–5.28)*	3.93 (3.49–4.43)*	3.30 (2.89–3.77)*	3.11 (2.79–3.46)*	3.56 (2.56–4.96)*
Sleep apnea	1.56 (1.51–1.60)*	1.63 (1.57–1.69)*	2.10 (1.97–2.23)*	1.57 (1.47–1.68)*	1.99 (1.90–2.08)*	1.38 (1.09–1.75)**
*c*-statistic	0.68	0.73	0.69	0.77	0.73	0.80

CI: confidence interval. *P < 0.001, **P < 0.01, and ***P < 0.05.

**Table 5:** This table shows the PAF for risk factors on selected outcomes.

Exposure	Population attributable fraction (95% CI)					
	Combined major complications	Major cardiac complications	Major pulmonary complications	Acute renal failure	30-Day mortality	Critical care services admission
Blood transfusion	9.51% (9.12–9.90)	4.77% (4.22–5.32)	19.64% (18.41–20.86)	29.75% (28.41–31.08)	17.15% (12.71–21.37)	10.90% (10.08–11.73)
All comorbidities	13.40% (12.94–13.86)	12.42% (11.75–13.07)	21.46% (20.12–22.78)	32.77% (31.37–34.14)	20.66% (16.07–25.00)	19.44% (18.47–20.40)
Diabetes	3.17% (2.82–3.52)	2.03% (1.52–2.53)	2.55% (1.48–3.61)	11.86% (10.59–13.11)	5.21% (1.46–8.81)	2.03% (1.28–2.77)
Sleep apnea	3.44% (3.20–3.68)	4.23% (3.88–4.58)	8.47% (7.64–9.30)	5.62% (4.73–6.50)	2.50% (0.08–4.85)	7.35% (6.77–7.93)
Obesity	4.19% (3.85–4.53)	2.59% (2.14–3.05)	7.47% (6.37–8.55)	15.94% (14.73–17.13)	3.78% (0.63–6.83)	7.07% (6.29–7.84)
Age group* (ref ≤65)	34.70% (33.63–35.75)	58.02% (56.85–59.16)	15.92% (12.23–19.46)	19.46% (15.43–23.30)	49.54% (38.69–58.47)	11.37% (8.78–13.89)
Gender*(ref= F)	8.18% (7.63–8.72)	19.73% (18.93–20.52)	2.76% (1.15–4.34)	22.73% (21.04–24.37)	23.69% (18.07–28.92)	6.03% (4.85–7.20)

CI: confidence interval of the PAF.

*PAF compares the proportion of outcome to a population, where all individuals are ≤65 years or are all female for the risks age group and gender, respectively.

**Table 6:** International classification of diseases-9th revision-clinical modification (ICD-9-CM) codes for comorbidities and complications.

Measure	ICD-9-CM codes
Pulmonary hypertension	416.X
Sleep apnea	786.03, 780.51, 780.53, 780.57, 327.20–327.27, 327.29
Pulmonary embolism	415.1
Deep vein thrombosis	451.1, 451.2, 451.8, 451.9, 453.2, 453.4, 453.8, 453.9
Cerebrovascular event	433.01, 433.11, 433.21, 433.31, 433.81, 433.91, 434.01, 434.11, 434.91, 997.02
Pulmonary compromise	514, 518.4, 518.5, 518.81, 518.82
Sepsis	038, 038.0, 038.1x, 038.2, 038.3, 038.40, 038.41, 038.42, 038.43, 038.44, 038.49, 038.8, 038.9, 790.
Cardiac (Nonmyocardial Infarction)	426.0, 427.41, 427.42, 429.4, 997.1, 427.4, 427.3, 427.31, 427.32
Acute myocardial infarction	410.XX
Pneumonia	481, 482.00–482.99, 483,485, 486, 507.0, 997.31, 997.39
All Infections	590.1, 590.10, 590.11, 590.8, 590.81, 590.2, 590.9, 595.0, 595.9, 599.0, 567.0 480, 480.0, 480.1, 480.2, 480.8, 480.9, 481, 482.0, 482.1, 482.2, 482.3, 482.30, 482.31, 482.32, 482.39, 482.4, 482.40, 482.41, 482.42, 482.49, 482.5, 482.8, 482.81, 482.82, 482.83, 482.84, 482.89, 482.9, 483, 483.0, 483.1, 483.8, 485, 486, 487, 997.31, 038, 038.0, 038.1, 038.10, 038.11, 038.12, 038.19, 038.2, 038.3, 038.4, 038.40, 038.41, 038.42, 038.43, 038.44, 038.49, 038.8, 038.9, 790.7, 998.0, 958.4, 998.5, 998.59, 998.89, 785, 785.50, 785.52, 785.59, 999.39, 999.31, 999.3
Acute renal failure	584, 584.5, 584.9
Gastrointestinal complication	997.4, 560.1, 560.81, 560.9, 536.2, 537.3

**Table 7:** This table shows the PAF for risk factors on various references of age group.

Outcome	Population attributable fraction (95% CI)					
	Combined major complications	Major cardiac complications	Major pulmonary complications	Acute renal failure	30-Day mortality	Critical care services admission
Age group (ref ≤45)	55.94% (52.89–58.80)	84.22% (81.53–86.52)	39.38% (28.39–48.68)	49.96% (38.65–59.19)	72.41% (37.91–87.74)	11.22% (2.31–19.32)
Age group (ref ≤55)	48.94% (47.47–50.36)	75.45% (74.03–76.79)	28.78% (23.54–33.66)	38.71% (33.27–43.71)	59.13% (43.52–70.43)	10.70% (6.68–14.56)
Age group (ref ≤65)	34.70% (33.63–35.75)	58.02% (56.85–59.16)	15.92% (12.23–19.46)	19.46% (15.43–23.30)	49.54% (38.69–58.47)	11.37% (8.78–13.89)
Age group (ref ≤75)	16.80% (16.29–17.30)	27.80% (27.04–28.55)	11.35% (9.85–12.83)	13.96% (12.33–15.56)	38.78% (33.31–43.81)	11.09% (10.07–12.10)

CI: confidence interval of the PAF.

*PAF compares the proportion of outcome to a population where all individuals are *less than the specified*
